# Investigating artificial intelligence models for predicting joint pain from serum biochemistry

**DOI:** 10.1590/1806-9282.20240381

**Published:** 2024-09-16

**Authors:** Saman Shahid, Aatir Javaid, Usman Amjad, Jawad Rasheed

**Affiliations:** 1 National University of Computer and Emerging Sciences, Foundation for the Advancement of Science and Technology, Department of Sciences and Humanities – Lahore, Pakistan.; 2 The University of Lahore, University College of Medicine, Department of Orthopedic Surgery – Lahore, Pakistan.; 3 Istanbul Sabahattin Zaim University, Department of Computer Engineering – İstanbul, Turkey.

**Keywords:** Joint pain, Arthritis, Random forest, Perceptrons, C-reactive protein, Uric acid, Creatinine

## Abstract

**OBJECTIVE:**

The study used machine learning models to predict the clinical outcome with various attributes or when the models chose features based on their algorithms.

**METHODS:**

Patients who presented to an orthopedic outpatient department with joint swelling or myalgia were included in the study. A proforma collected clinical information on age, gender, uric acid, C-reactive protein, and complete blood count/liver function test/renal function test parameters. Machine learning decision models (Random Forest and Gradient Boosted) were evaluated with the selected features/attributes. To categorize input data into outputs of indications of joint discomfort, multilayer perceptron and radial basis function-neural networks were used.

**RESULTS:**

The random forest decision model outperformed with 97% accuracy and minimum errors to anticipate joint pain from input attributes. For predicted classifications, the multilayer perceptron fared better with an accuracy of 98% as compared to the radial basis function. Multilayer perceptron achieved the following normalized relevance: 100% (uric acid), 10.3% (creatinine), 9.8% (AST), 5.4% (lymphocytes), and 5% (C-reactive protein) for having joint pain. Uric acid has the highest normalized relevance for predicting joint pain.

**CONCLUSION:**

The earliest artificial intelligence-based detection of joint pain will aid in the prevention of more serious orthopedic complications.

## INTRODUCTION

Machine learning (ML) is rapidly being applied in numerous disciplines of medicine for illness detection, stratification, and prediction in both at-risk groups and diseases^1^. Joint redness, soreness, swelling, limping, freezing, weakness, etc. are the common signs of joint pain. Arthritis affects 24% of all adults in the United States. It is the biggest cause of work disability^2^. In Pakistan, the average age of osteoarthritis patients is 58 years old. Women are more likely to suffer from osteoarthritis. Diabetes, hypertension, obesity, cardiovascular disease, chronic renal failure, use of diuretics, a diet high in purine-rich foods, and alcohol usage are risk factors for gout-related joint discomfort and swelling^3^. A variety of ailments, including infections and autoimmune diseases like rheumatoid arthritis and inflammatory bowel disease, can result in inflammation. Increased CRP in the presence of joint pain suggests the probability of autoimmune arthritis or joint infection^4^.

We included a variety of models to evaluate how artificial intelligence (AI) interfaces in predicting clinical outcomes when we train the models with different attributes or when models pick features depending on their algorithms. Early detection of joint pain can help prevent more serious complications like arthritis, bursitis, fibromyalgia, osteoarthritis, tendinitis, and so on. For this purpose, we included those subjects who were suffering from mild joint edema or myalgia and visited the orthopedic department. We tested two ML decision trees (random forest, and gradient boosted) to report and compare their efficiencies in predicting the target variable (joint pain) based on the clinical biochemistry data of individuals. Clinical variables such as risk factors (such as uric acid, CRP, etc.) were taken to predict the diagnostic outcome for the indication of joint pain in the patients. We also employed multilayer perceptron (MLP) and radial basis function (RBF) to match the results and determine the relative percentage of normalized relevance of each clinical/biochemistry variable in influencing joint pain. Decision trees are nonparametric supervised learning approaches used in ML for classification^5^. Artificial neural networks (ANNs) research is available that can focus on the biomechanics of the spine by elucidating joint moments, spinal stresses, and muscle forces. Zeng et al.^6^ created a prediction model to predict the risk of hyperuricemia in Chinese individuals. Machine learning fills a big void in clinical experience learning, diagnosing predictions, and advising accurate treatment. Therefore, the primary goal was to determine if decision trees constructed using machine learning techniques and neural networks could predict joint pain based on biochemical data.

## METHODS

A cross-sectional study was conducted after ethical approval from the University of Lahore (UOL), Mansoorah Teaching Hospital, Lahore. The study was conducted from January 2022 to June 2022. A total of 650 patients were included. This determined that machine learning decision models and neural networks can correctly predict joint pain under given biochemistry variables. Patients aged 20–65 years who visited outpatient department (OPD) with moderate joint swelling or myalgia were included. Cases of severe comorbidities, autoimmune, or degenerative joint conditions were not included. Through a predesigned proforma, the basic and clinical data were collected. The patients were also inquired about the presence of joint pain, which was documented on proforma. Clinical laboratory findings were conducted at the affiliated UOL's Mansoorah Hospital, Lahore. A chi-square test was applied to see the significant or insignificant difference between the expected and observed values of variables.

ML models Random Forest and Gradient Boosted were evaluated. The attributed dataset of outdoor patients was included to build the models. Observations about a parameter are provided in branches of a predictive decision tree model, which leads to inferences about the targets of the parameter. Sorting the other examples from the tree's root to the leaf nodes allowed for the categorization of each instance. Gender, age, uric acid, and CRP are considered attributes representing risk factors, and outcome in terms of identification of joint pain was considered the target variable.

A random forest operator was used to create a random forest model for classification. These trees were built or trained using bootstrapped subsets of the dataset sent over the input. A tree node represented a splitting rule for a single attribute. For splitting rule selection, only a subset of attributes determined by the subset ratio requirement was evaluated. The process of creating additional nodes was repeated until the halting requirements were fulfilled. Pruning was used to simplify the model by substituting leaves for sub-trees that had weak prediction abilities^5^. Gradient Boosted was utilized for trees that run the gradient boosted (GBT) algorithm using H2O 3.30.0.1. For the prediction of the labeled attribute, this classification model was used for data sets^5^.

The joint pain indication was labeled as yes or no. To build the model, the data sets were accessed, identified the target output, assigned the training (70%) and testing (30%) portions, trained the model under supervised learning methods, generated the model, and evaluated the performance variables (accuracies and errors) with cross-validations (10-fold).

ANN MLP model was developed to predict the presence or absence (as an outcome) of joint discomfort by evaluating data from recorded scaled variables. We began with the input layer and worked our way forward to the output layer while creating MLP. The error was determined based on the output. These stages were repeated numerous times to learn the optimal weights. The result was created using functions to predict the designated class. A four-layered ANN, through a supervised learning algorithm with batch training, was generated to classify input data (factors and covariates) into output categories as clinical outcomes. Of this, 70.2% of the data were segregated as a training set, and 29.8% were taken as a testing set. The stopping rule was used for training. Biochemistry parameters, along with age and gender, were used as predictor variables to predict dependent variable outcomes. We used the "standardized" rescaling method of covariates for the input layer. A technique for supervised back-propagation learning was used to train the MLP model. To control the synaptic weights, a batch training mode with a scaled conjugate gradient was chosen. We used the following values: 0.4 starting learning rate, 0.9 momentum, 0 interval center, and 0.5 interval offset. The use of linear combinations of terms based on a single univariate function to approximate multivariable functions is known as the radial basis function. We incorporated the radial basis-neural network (RB-NN) by adding the radial basis function as a neuron and compared the input data to the training data. The percentage normalized importance was calculated with all independent predictor variables for both models.

## RESULTS

**Figure 1:**
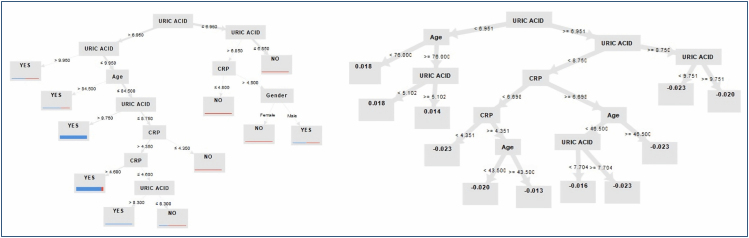
The decision trees analyze the uric acid first (as a root node) with values above or below the normal range. If uric acid is found above the normal range, the decision model looks for the age factor, and with increased age, it evaluates the C-reactive protein values (above or below the normal range), etc. for the prediction of joint pain (as leaf nodes) in yes/no for random forest, and leaf nodes (average of all residuals for the specified loss function) as the final output (joint pain) prediction, comprising the sum of all previous predictions and the overall prediction of the current tree.

The chi-square test showed significant differences (p-value<0.050) between the expected and observed values in the following variables: hematocrit, eosinophils, CRP, blood urea nitrogen, creatinine, total bilirubin, ALT, AST, and ALP (Table 1). Random forest and gradient-boosted models both achieved 97.44% accuracy with 96.92 and 96.77% accuracy on validation. Although the percentage accuracy was the same at 97.44% in the gradient-boosted model; however, its relative errors were quite greater as compared to the random forest model. The random forest decision model performed best (Figure 1).

**Table 1:** Clinical statistics.

Parameter	Yes	No	p-value
Joint pain	295 (45.3%)	355 (54.6%)	0.004
Variables	Min./Max.	Mean±SD	p-value
Age (years)	20/85	52.56±18.63	0.776
Uric acid. NR§: 3.5–7.0 mg/dL	3/10	6.57±1.97	0.469
Hemoglobin. NR: 12–18 g/dL	9/14	11.48±1.44	0.072
WBC. NR: 4–11 × 10^3^	4.10/13.80	8.57±2.59	0.487
Neutrophils. NR: 45–70%	45/82	60.00±9.18	1.00
Lymphocytes. NR: 25–45%	25/50	37.22±7.39	1.00
Red blood cells (RBC). NR: 4.5–6.5 million/mm^3^	2/14.50	9.63±2.95	0.103
Mean cell volume. NR: 85–95%	85/100	92.59±4.34	0.988
Mean corpuscular hemoglobin. NR: 27–32 pg	27/32	29.36±1.45	0.086
Mean corpuscular hemoglobin concentration. NR§: 30–35 g/dL	30/35	32.52±1.47	0.071
Hematocrit (HCT). NR: 48–55%	48/55	51.55±2.00	0.030
Eosinophil NR: 1–6%	1/6	3.56±1.51	0.000
C-reactive protein (CRP). NR: up to 10 mg/L	3/14.10	6.47±2.06	0.000
Erythrocyte sedimentation rate (ESR). NR: up to 10 mm/1st h	7/15	10.97±2.32	0.115
Blood urea nitrogen (BUN). NR: 10–50 mg/dL	2/65	36.30±14.93	0.000
Creatinine. NR: 0.5–1.2 mg/dL	0.1/1.90	0.915±0.24	0.000
Total bilirubin. NR: up to 1.0 mg/dL	0/3	0.928±0.407	0.000
Alanine transaminase (ALT). NR: 5–42 U/L	3/84	38.96±9.07	0.000
Aspartate aminotransferase (AST). NR: 5–31 U/L	3/85	37.93±8.51	0.000
Alkaline hosphatase (ALP). NR: 98–279 U/L	39/383	199.41±59.11	0.000

§ NR: normal range.

**Figure 2:**
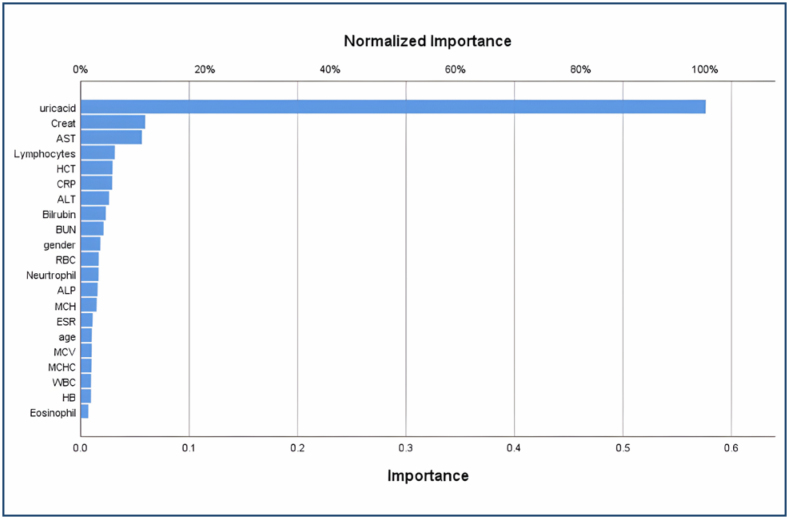
Independent variable importance chart.

The multilayer perceptron-neural network (MLP-NN) model produced just 2% inaccurate predictions for training and 4.6% for testing datasets, whereas the radial basis function-neural network (RBF-NN) model yielded 7.8% (training) and 7% (testing). For predicted classifications, the MLP-NN achieved 98% for training and 95.4% for testing datasets, whereas the RBF-NN showed 92.2% for training and 93% for testing datasets. The MLP model showed better performance and accuracy as compared to the RBF model. MLP and RBF both predicted the same percentage for hematocrit (HCT) (5.1%), red blood cells (2.9%), age (1.7%), and uric acid (100%). In all other biochemistry variables (except lymphocytes), RBF predicted more normalized importance percentages than MLP. Model MLP achieved the following percentages: 10.3% (creatinine), 9.8% (AST), 5.4% (lymphocytes), and 5% (CRP) for having joint pain. RBF yielded 24.5% (creatinine), 19.8% (AST), 3.2% (lymphocytes), and 8.2% (CRP) for joint pain. The analysis concludes that uric acid has the highest normalized importance for the anticipation of joint pain (Figure 2).

## DISCUSSION

It is known that uric acid is linked with the indication of joint pain. With 97% accuracy and minimal errors, the random forest decision model outscored all others. Regarding neural networks, MLP obtained 98% for training and 95.4% for testing datasets for predicted classifications. Based on both MLP and RBF results, the research suggests that uric acid has the highest normalized relevance for the prediction of joint discomfort. ANN and ML systems can provide reliable and optimum performance for clinical diagnosis, prognosis, and personalized therapy by employing mathematical algorithms and computational approaches. Researchers developed methods to detect the earliest pain progression to avoid knee osteoarthritis. Researchers have applied machine learning-based models that anticipated knee pain with 84% accuracy. They offered a technique that can identify the advancement of pain early on, hence enhancing future attempts to avoid knee osteoarthritis^7^. Recently, the systemic risk factors were evaluated in knee-osteoarthritis onsets from various combinations of the DeepLIFT gradient. Their methods predicted diabetes and smoking as the highest gradient factors^8^. Another study employed a support vector machine with 74% accuracy to predict knee-osteoarthritis progression^9^. Abnormal joint moments during walking are proven predictors of osteoarthritic knee discomfort. In another experiment, four machine-learning methods were utilized. The random forest method fared best in terms of prediction performance, but it was also the slowest by a factor of ten. The multilayer perceptron neural network technique outperformed the others in terms of root mean square error and area under the recall curve^10^. Chronic impairment in temporomandibular joint osteoarthritis worsens with age, and the primary objective is to detect the disease before morphological degradation develops. Bianchi et al.^11^ tackled this difficulty by processing and analyzing 52 clinical, biochemical, and high-resolution cone beam computed tomography (CBCT) (radiomics) variables from temporomandibular joint osteoarthritis patients and controls using sophisticated data science. They included machine-learning models with good accuracy for diagnosing temporomandibular joint osteoarthritis. In a study, deep neural networks were found effective for the classification of the complicated problem of knee osteoarthritis diagnosis. Gender, age, and obesity were used to categorize the subgroups studied^12^. Hafezi-Nejad et al.^13^ investigated the relationship between baseline cartilage volume measures and medial compartment joint space-loss advancement. The most relevant estimators for medial joint-space-loss progression in the MLP-ANN studies were baseline and interval changes in lateral-femoral cartilage volume. K-means algorithms are also used to predict rheumatoid arthritis patients with 84% accuracy^14^. Soin et al.^15^ achieved 72% accuracy in detecting spinal pain and concluded that AI decision tools are quite effective. ML models should ideally integrate acquired information into clinical evidence, with computers capable of predicting clinical outcomes, identifying illness patterns, detecting disease signs, and improving treatment procedures^16^.

## CONCLUSION AND RECOMMENDATIONS

The random forest decision model performed better overall in anticipating joint pain with biochemistry parameters. Uric acid has the highest normalized relevance for predicting joint pain. The platforms of AI models should be used in hospitals with integrated computerized medical systems.

## ETHICAL APPROVAL

The study was conducted in accordance with the Declaration of Helsinki and followed the ethical standards of Pakistan. The study was conducted after getting ethical approval from the hospital's ethical review committee. The date of approval was March 3, 2022. The study conformed to institutional ethical standards.
